# Geriatric nutritional risk index as a predictor of prognosis in hematologic malignancies: a systematic review and meta-analysis

**DOI:** 10.3389/fnut.2023.1274592

**Published:** 2023-10-24

**Authors:** Qiong Yu, Mengxing Tian, Guoliang Pi, Yegui Jia, Xin Jin

**Affiliations:** ^1^Department of Digestive Medicine, Wuhan Sixth Hospital and Affiliated Hospital of Jianghan University, Wuhan, China; ^2^Department of Clinical Nutrition, Hubei Cancer Hospital, Tongji Medical College, Huazhong University of Science and Technology, Wuhan, China; ^3^Department of Thoracic Oncology, Hubei Cancer Hospital, Tongji Medical College, Huazhong University of Science and Technology, Wuhan, China

**Keywords:** geriatric nutritional risk index, diffuse large B-cell lymphoma, hematologic malignancies, overall survival, meta-analysis

## Abstract

**Objective:**

Recent studies have reported inconsistent results regarding the association between geriatric nutritional risk index (GNRI) and clinical outcomes in patients with hematologic malignancies (HMs). We performed a meta-analysis to evaluate the effect of low GNRI on the overall survival (OS) and progression-free survival (PFS) in patients with HMs.

**Research Methods and Procedures:**

We conducted the research via PubMed, Embase, and Cochrane Library databases to identify trials. Exploring the association between GNRI and prognosis in patients with HMs. A meta-analysis of OS and PFS was performed. Quality In Prognostic Studies instrument and Newcastle–Ottawa quality assessment Scale were used to assess the quality of included trials.

**Results:**

Fourteen studies enrolling 3,524 patients with HMs were included. Low GNRI was associated with shorter OS (Hazard ratio (HR) = 1.77; 95% CI = 1.44–2.18, *p* < 0.01) and PFS (HR = 1.63; 95% CI = 1.17–2.27, *p* < 0.01) in patients with HMs. In the subgroup analysis, GNRI was not significantly associated with prognosis in Chinese patients with HMs (OS, HR =1.33; 95% CI = 0.89–1.98, *p* = 0.16; PFS, HR = 1.70; 95% CI = 0.72–4.01, *p* = 0.23). For the subgroup with a GNRI cutoff value less than 98, there was no significant difference in PFS (HR = 1.34; 95% CI = 0.98–1.83, *p* = 0.06).

**Conclusion:**

Low GNRI negatively impacted on the prognosis in patients with HMs. Prospective studies to identify the best cut-off value for GNRI are required.

## 1. Introduction

Hematologic malignancies (HMs), also called blood cancers, are a diverse group of cancers originating from blood-forming tissue or cells of the immune system. There are three main types of hematologic tumors, lymphoma, myeloma, and leukemia. In 2022, there were approximately 184,130 new cases and 57,810 new deaths of hematologic malignancies in the US estimated by the North American Association of Central Cancer Registries and US mortality data (1). Even though much progress has been made in personalized therapy and many new drugs have been developed for HMs in recent years (2), the prognosis of HMs is still poor for some patients, with a 5-year survival ranging from 24 to 86% (3). Immunotherapies, including checkpoint inhibitors and therapeutic vaccines, have also been applied to HMs to improve patient survival (4, 5). However, chemotherapy, radiotherapy and immunotherapies do not achieve satisfactory therapeutic effects for all cancer types and patients with HMs (4). Various prognostic factors based on clinical and pathologic features such as stage of disease, histological subtype, positron emission tomography (PET) imaging, tissue and circulating biomarkers and gene expression profiling have been widely used to predict hematologic malignancies survival (6–9). However, those prognostic factors are not enough for predicting prognosis precisely. In addition, detecting genetic markers is costly and is not readily available in clinical practice in the low-income group.

Multiple studies have confirmed that baseline nutrition status is a common factor associated with decreased overall survival time in patients with HMs undergoing chemotherapy and allogeneic hematopoietic cell transplantation (HCT) (10–14). Moreover, it has been proposed that nutritional treatment would improve the prognosis in patients with HMs before HCT (15). Nevertheless, there are no consensus diagnostic criteria for malnutrition in patients with HMs. Hence, it is crucial to identify an effective nutrition index to predict the prognosis of patients with HMs.

GNRI is a simple and accurate nutritional index invented to predict morbidity and mortality in hospitalized elderly patients (16). GNRI consists of serum albumin levels and the ideal body weight, calculated using the Lorentz formula [1.489 × serum albumin (g/L)] + [41.7 × (current body weight/ideal body weight)]. The height and sex-specific formulas were used to calculate ideal body weight. For GNRI, studies have confirmed its good predictability of clinical outcomes for patients with acute ischemic stroke (17), polytrauma (18), heart failure (19), undergoing emergency surgery (20), and receiving hemodialysis (21). Subsequent studies have verified the prognostic value of GNRI of patients with solid cancers (22–24). Recently, emerging evidence has reported the prognostic significance of pretreatment GNRI in patients with HMs undergoing chemotherapy, immunotherapy and HCT. However, those studies have garnered inconsistent results (25–27). Some studies showed that low GNRI was associated with poorer prognosis in patients with diffuse large B-cell lymphoma (DLBCL), myelodysplastic syndrome (MDS) and acute myeloid leukemia (AML) (28, 29). However, Li’s study suggested that GNRI was not an independent predictor in patients with DLBCL (30). Two previously published meta-analyzes, which reviewed the predictive role of GNRI in DLBCL, both found that low GNRI was associated with shorter survival. However, only seven articles were included. In the results analyzing the relationship between GNRI and PFS, only 2 and 3 studies were included, respectively, which would lead to inaccurate results (31, 32). To date, we found that other five new studies (27, 29, 33–36) also explored the relationship between GNRI and survival outcomes in patients with HMs. To our knowledge, no meta-analyzes have been conducted on the potential prognostic value of GRNI in patients with non-DLBCL HMs. Therefore, we conducted a meta-analysis to summarize emerging evidence regarding the prognostic value of GRNI in patients with HMs.

## 2. Materials and methods

### 2.1. Search strategy and selection criteria

The methods of the present meta-analysis were performed following the Meta-Analyzes (PRISMA) statement. We searched the PubMed, Embase, and Cochrane Library databases for all references from January 2005 to July 2023. The keywords and MeSH terms “hematologic malignancies,” “Geriatric Nutritional Risk Index” and other related words were used in the search process. The search strategy was [GNRI(All Fields) OR “Geriatric nutritional risk index” (All Fields)] AND [“leukemia” (All Fields) OR “lymphoma” (All Fields) OR “myeloma” (All Fields) OR “hematologic malignancy” (All Fields) OR “hematopoietic malignancy” (All Fields) OR “hematopoietic neoplasms” (All Fields) OR “hematological malignancy” (All Fields) OR “hematological neoplasms” (All Fields) OR “hematologic neoplasms” (All Fields) OR “multiple myeloma” (All Fields) OR “diffuse large B cell lymphoma” (All Fields)]. Two independent researchers searched twice to avoid omissions in the literature search. The references and conference articles were also reviewed.

### 2.2. Study selection and data extraction

Studies were included if (1) the study was designed as a prospective cohort study, a randomized controlled trial, or a retrospective study; (2) the subjects were patients diagnosed with any hematologic malignancies; (3) GNRI was calculated before or during the treatment, (4) the outcomes included OS and PFS. Studies published in abstract form, case reports, and non-English literature were excluded.

Two authors extracted data independently from eligible articles; another independently checked all extracted data. The following details were included: the name of the first author, the year of publication, characteristics of study participants, numbers of participants, the cut-off value of GNRI, treatment methods for hematologic malignancies, median OS, and outcomes.

### 2.3. Quality assessment

The quality assessment of included trials was performed independently using the Quality In Prognostic Studies tool by two reviewers. This tool consists of six domains: study participation, study attrition, prognostic factor measurement, outcome measurement, study confounding, and statistical analysis and reporting. If more than four of these six domains are at low risk of bias, the overall risk of bias of the literature is low; if two or more than two domains are at high risk of bias, the overall evaluation of literature quality is high, and the remaining studies were classified as the moderate risk of bias (37). The Newcastle–Ottawa quality assessment Scale consists of eight components divided into three areas. Each research received a maximum of nine points. The tool classifies articles with a total score of 6 or more as high-quality, articles with a score of 3 to 6 as intermediate, and articles with a score of less than 3 as poor quality.

### 2.4. Data synthesis and statistical analysis

The Stata software (version 14) was used for statistical analysis. Cochran’s Q and I squared tests were performed to identify between-study heterogeneity for each analysis. *I*^2^ < 25% was defined as no heterogeneity, *I*^2^ = 25 ~ 50% moderate heterogeneity, and *I*^2^ > 50% considerable heterogeneity. When heterogeneity across studies was considerable, the random-effects model was chosen to calculate the pooled hazard ratio (HR) and the corresponding 95% CIs. Otherwise, the fixed-effects model would be used. HR was defined as risk of outcome in low GNRI group/ risk of outcome in high GNRI group. Most of the adjusted HRs values were extracted from multivariate Cox regression analyzes. If the multivariate analysis was not carried out in the study, the outcomes from univariate Cox regression were used for analysis. A few HRs and 95% CIs were recalculated from Kaplan–Meier curves. A predefined subgroup analysis based on the country, cut-off value, the number of participants, median age, type of HMs and median OS was performed to explore the prognostic role of GNRI further. To further explore the prognostic value of GNRI on lymphoma, a separate pooled analysis and meta regression were performed for studies in which all the participants were lymphoma. The sex (% of males), age (% of median age < 70), stage (% of stage I – II), Eastern Cooperative Oncology Group performance status score (ECGO PS) (% of 0–1), B symptoms (% of absent), lactic dehydrogenase (LDH) (% of normal), country, cut-off value (% of >98), the number of participants (% of number > 200), and median OS(% of OS <48 month) were used as potential moderators to conduct the meta-regression using univariate model. Potential publication bias was judged by funnel plots and the Egger test.

## 3. Results

### 3.1. Study characteristics

**Figure 1:**
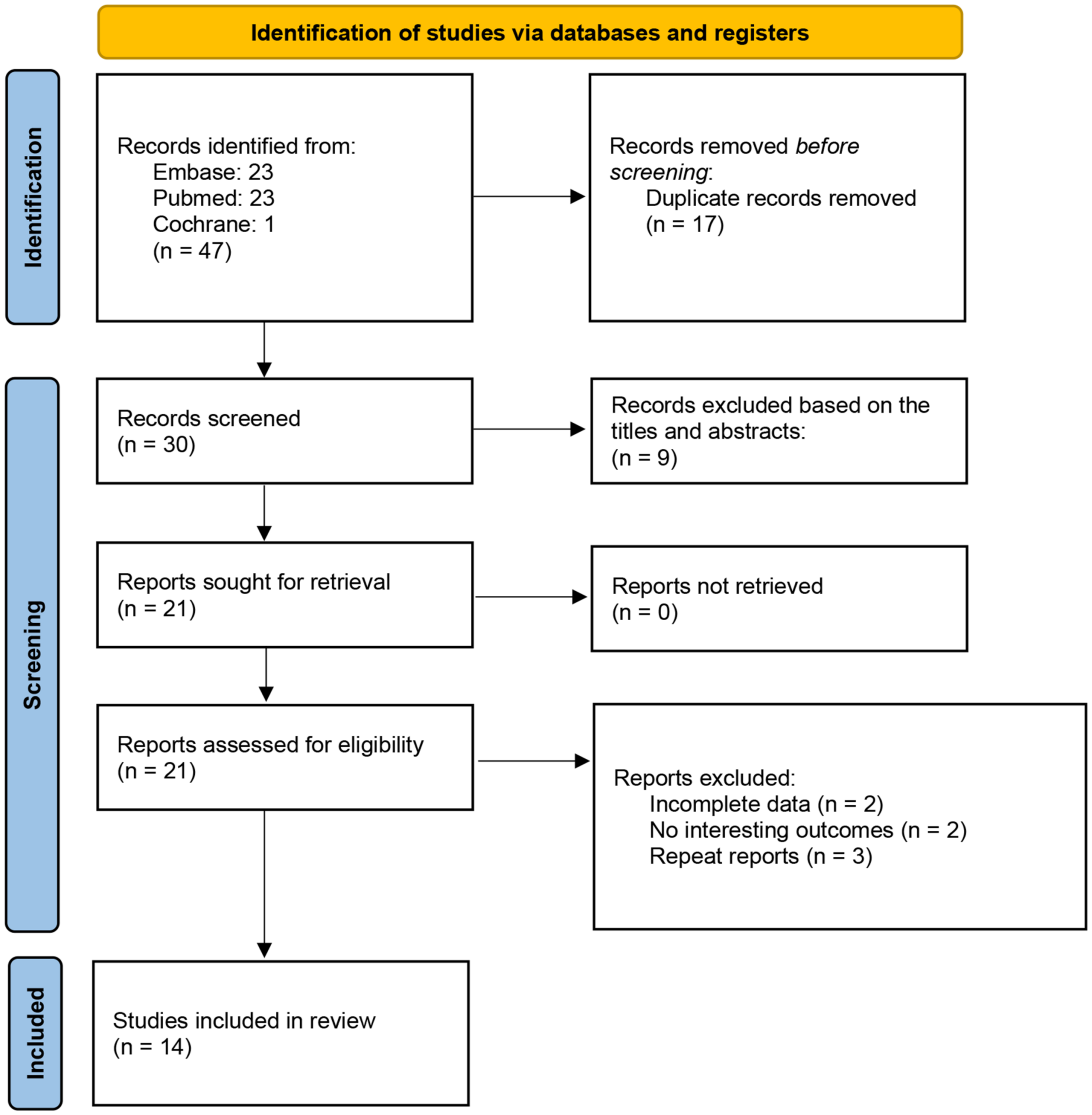
A flow chart of the study selection process.

The selection process was summarized in a PRISMA flowchart (Figure 1). Firstly, 47 potential studies were obtained from databases and other sources during the search process. Seventeen studies were excluded due to duplication. Another nine articles were excluded after screening titles and abstracts. Then 21 studies were examined by reviewing the full texts, and seven studies were excluded since they had no interesting results or incomplete data. Ultimately, 14 studies with 15 comparisons met the eligibility criteria and were included.

The baseline characteristics of the included studies are shown in Table 1. Thirteen studies were retrospective and one study was prospective (35). The number of participants in the included studies ranged from 86 to 615. A total of 3,524 patients were enrolled in the 14 studies. Participants in 10 included studies were patients with DLBCL (25, 26, 28, 30, 35, 36, 38–41). Participants in two studies were diagnosed with lymphoma (33, 34), and participants in the other two were diagnosed with myeloid leukemia (27, 29). The cut-off value of GNRI ranged from 92 to 106.26.

**Table 1:** Characteristics of the trials included in the meta-analysis.

Study, year	Country	*n* (Male/Female)	Age (range, median)	Population	GNRI cutoff value	Treatment	median OS (month)	Outcomes
Dongmei Yan 2021	China	67/66	60–91,71	DLBCL	106.26	R-CHOP	19.4	OS
Se-Il Go 2020	Korea	130/98	21–88,64	DLBCL	82,92,98	R-CHOP	89.4	OS PFS
Shin Lee 2021	Japan	223/228	65–96,78	DLBCL	98	R-CHOP/non-standard therapies	22.3	OS
Toshihiro Matsukawa 2020	Japan	337/278	20–97,69	DLBCL	95.7	R-CHOP	not reached	OS
Tzer-Ming Chuang 2021	China	107/98	65–96,75	DLBCL	92.5	R–CHOP/ Non-R–CHOP	13.5	OS PFS
Unal Atas 2022	Turkey	112/94	NR,58.57	DLBCL	104.238	R–CHOP	116	OS
Yusuke Kanemasa 2018	Japan	266/210	27–97,68	DLBCL	96.8	R-CHOP/R-THP- COP	45	OS
Zhongqi Li 2018	China	156/111	47.25–63.75, NR	DLBCL	98	R–CHOP	not reached	OS
Yuriko Nishiyama-Fujita 2020	Japan	61/25	43–93,74	MDS and AML	93.82	azacytidine	12	OS
Akihito Nagata 2022	Japan	107/57	19–73,51	AML	92	first allo-HSCT	58.7	OS PFS
Kota Mizuno 2019	Japan	60/70	32–91,67	follicular lymphoma	99.2	NR	52	OS
Ken Kikuchi 2023	Japan	61/70	69–83, 77	Lymphoma	92	R–CHOP	NR	PFS (TTF)
Pénichoux Juliette, 2023	France	47/48	78.4(mean)	DLBCL	82,92,98	R–CHOP/R-miniCHOP	22.7	OS PFS
Yu Yagi 2023	Japan	183/154	70–97, 70	DLBCL	82,92	chemotherapy	60.1	OS PFS

GNRI, Geriatric nutritional risk index; DLBCL, Diffuse large B-cell lymphoma; MDS, Myelodysplastic syndromes; AML, Acute myeloid leukemia; R-CHOP, Retuximab, cyclophosphamide, doxorubicin, vincristine, and prednisone; OS, Overall survival; PFS, Progression-free survival; TTF, time to treatment failure; allo-HSCT, allogeneic hematopoietic cell transplantation; R-THP- COP, Pirarubicin, cyclophosphamide, vincristine, and prednisolone; NR, Not reported.

### 3.2. Risk of bias of individual studies

The details of the risk of bias are listed in supplemental file, accessed by the QUIPS tools. Three trials had an overall low risk of bias, and one studies had an overall high risk of bias. The rest had an overall moderate risk of bias. While based on the score of Newcastle–Ottawa quality assessment Scale, there were none trials for poor quality, 11 trails for intermediates, and three trials for high quality.

### 3.3. Meta-analysis results for OS

**Figure 2:**
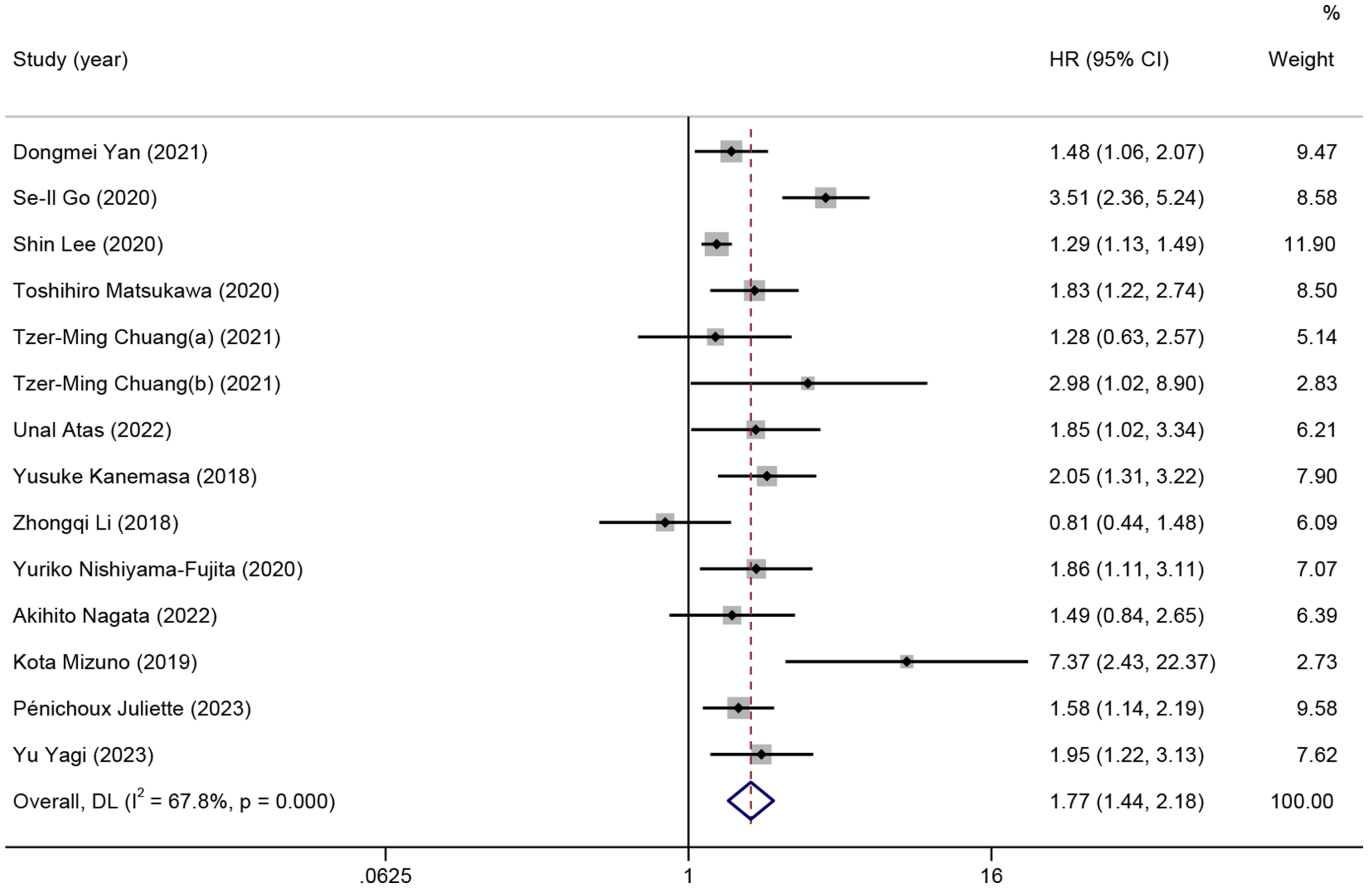
The forest plot for the association between GNRI and overall survival. The boxes and the horizontal lines through the boxes illustrate the estimated hazard ratios and the length of the confidence interval. The diamond and horizontal line through the diamond show the pooled hazard ratio and confidence interval.

In total, 13 studies reported HRs for OS of patients with HMs. As significant heterogeneity was detected, the random effects model was used (*I*^2^ = 67.8%). Patients with low pretreatment GNRI tended to have a shorter OS than those with high GNRI (HR = 1.77; 95% CI = 1.44–2.18, *p* < 0.01; Figure 2).

Subgroup analysis was performed to comprehensively evaluate the relationship between GNRI and OS in patients with HMs. In the subgroup analysis based on the type of HMs, the observed significant survival time reduction was observed in patients with DLBCL (11 studies, HR =1.70; 95% CI = 1.37–2.12, *p* < 0.01; *I*^2^ = 68.9%) and patients with non-DLBCL (3 studies, HR = 2.33; 95% CI = 1.15–4.71, *p* = 0.02; *I*^2^ = 68.7%). In the subgroup analysis based on the region, low GNRI was not associated with poor OS in patients from China (4 studies, HR =1.33; 95% CI = 0.89–1.98, *p* = 0.16; *I*^2^ = 41.7%). In subgroups other than those above, all the pooled data indicated an association between low GNRI and shorter OS in patients with HMs (Table 2).

**Table 2:** Subgroup analysis of the association between GNRI and the overall survival of patients with HMs.

Subgroup	No. of comparisons	Hazard ratios (95% CI)	*p* value	Heterogeneity	
				I^2^	*p* value
Country					
Japan	7	1.81 (1.38–2.37)	**<0.01**	64.2%	0.01
China	4	1.33 (0.89–1.98)	0.161	41.7%	0.16
Other countries	3	2.18 (1.28–3.72)	**<0.01**	78.9%	<0.01
Number of patients					
>200	7	1.77 (1.44–2.18)	**<0.01**	80.2%	0.15
≤200	7	1.72 (1.33–2.23)	**<0.01**	36.4%	<0.01
Age (median)					
>70	7	1.46(1.27–1.67)	**<0.01**	12.5%	0.33
≤70	7	2.02(1.36–2.99)	**<0.01**	73.7%	<0.01
Cutoff value					
>98	7	1.77 (1.25–2.49)	**<0.01**	82.5%	<0.01
≤98	7	1.83 (1.50–2.23)	**<0.01**	0.0%	0.86
DLBCL vs. Non-DLBCL					
DLBCL	11	1.70 (1.37–2.12)	**<0.01**	68.9%	<0.01
Non-DLBCL	3	2.33(1.15–4.71)	**0.02**	68.7%	0.04
Lymphoma vs. AML&MDS					
Lymphoma	12	1.79 (1.42, 2.27)	**<0.01**	72.4%	<0.01
AML&MDS	2	1.68 (1.15, 2.47)	**<0.01**	0.0%	0.57
Median overall survival time					
<48 m	7	1.50 (1.28–1.75)	**<0.01**	22.0%	0.26
≥48 m	7	2.00 (1.34–2.98)	**<0.01**	73.8%	<0.01

HMs, hematologic malignancies; DLBCL, diffuse large B-cell lymphoma; CI, Confidence interval; AML, acute myeloid leukemia; MDS, myelodysplastic syndromes.

### 3.4. Meta-analysis results for PFS

**Figure 3:**
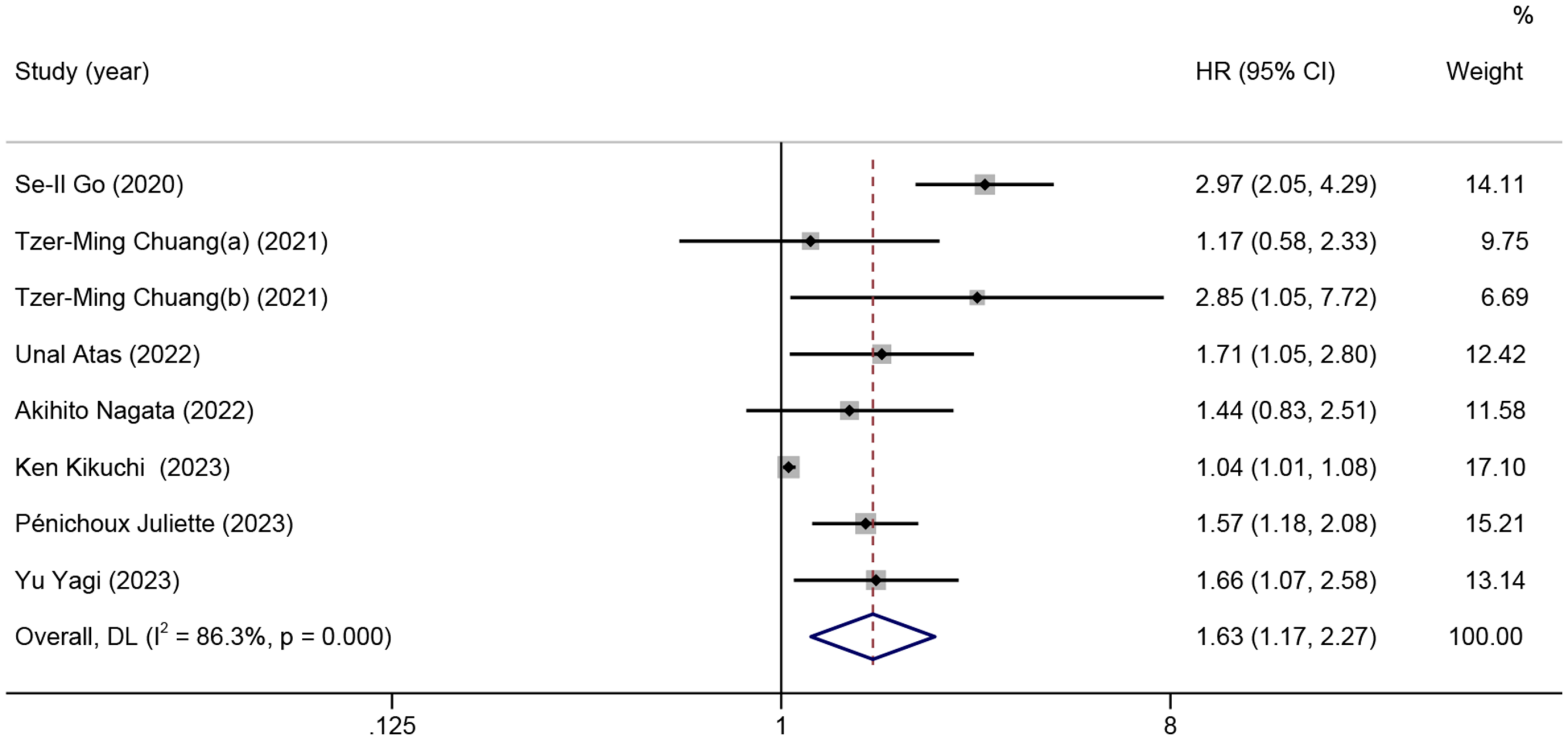
The forest plot for the association between GNRI and progression-free survival. The boxes and the horizontal lines through the boxes illustrate the estimated hazard ratios and the length of the confidence interval. The diamond and horizontal line through the diamond show the pooled hazard ratio and confidence interval.

Nine studies were included in the meta-analysis on the predictive value of GNRI on PFS. Patients with low GNRI had poorer PFS than patients with high GNRI (HR = 1.63; 95% CI = 1.17–2.27, *p* < 0.01), and significant heterogeneity was detected (*I*^2^ = 86.3%; Figure 3).

No significant association between PFS and GNRI was observed in the subgroup of patients from China (2 studies, HR = 1.70; 95% CI = 0.72–4.01, *p* = 0.23) and Japan (3 studies, HR = 1.27; 95% CI = 0.91–1.77, *p* = 0.17). For the subgroup with a cutoff value less than 98, there was no significant difference in PFS (5 studies, HR = 1.34; 95% CI = 0.98–1.83, *p* = 0.06). In the subgroup with non-DLBCL, no significant difference in PFS was observed for low GNRI vs. high GNRI (2 studies, HR = 1.08; 95% CI = 0.88–1.34, *p* = 0.46; Table 3).

**Table 3:** Subgroup analysis of the association between GNRI and the progression-free survival of patients with HMs.

Subgroup	No. of comparisons	Hazard ratios (95% CI)	*p* value	Heterogeneity	
				*I* ^2^	*p* value
Country					
Japan	3	1.27 (0.91–1.77)	0.17	64.4%	0.06
China	2	1.70 (0.72–4.01)	0.23	51.4%	0.15
Other countries	3	2.00 (1.31–3.03)	**<0.01**	73.3%	0.02
Number of patients					
>200	3	2.08 (1.40–3.10)	**<0.01**	60.7%	0.07
≤200	5	1.34 (0.99–1.82)	0.06	69.8%	0.01
Age (median)					
>70	5	1.40 (1.02–1.92)	**<0.05**	75.2%	<0.01
≤70	3	2.01 (1.27–3.18)	**<0.01**	65.4%	0.06
Cutoff value					
>98	3	2.00 (1.31–3.03)	**<0.01**	73.3%	0.02
≤98	5	1.34 (0.98–1.83)	0.06	58.4%	0.05
DLBCL vs. Non-DLBCL					
DLBCL	6	1.86 (1.41–2.45)	**<0.01**	51.5%	0.07
Non-DLBCL	2	1.08 (0.88–1.34)	0.46	24.5%	0.25
Lymphoma vs. AML&MDS					
Lymphoma	7	1.67 (1.16, 2.40)	**<0.01**	88.0%	<0.01
AML&MDS	1	1.44 (0.83, 2.50)	0.20	/	/

HMs, hematologic malignancies; DLBCL, diffuse large B-cell lymphoma; CI, Confidence interval; AML, acute myeloid leukemia; MDS, myelodysplastic syndromes.

### 3.5. Meta-analysis and meta-regression results for lymphoma

**Figure 4:**
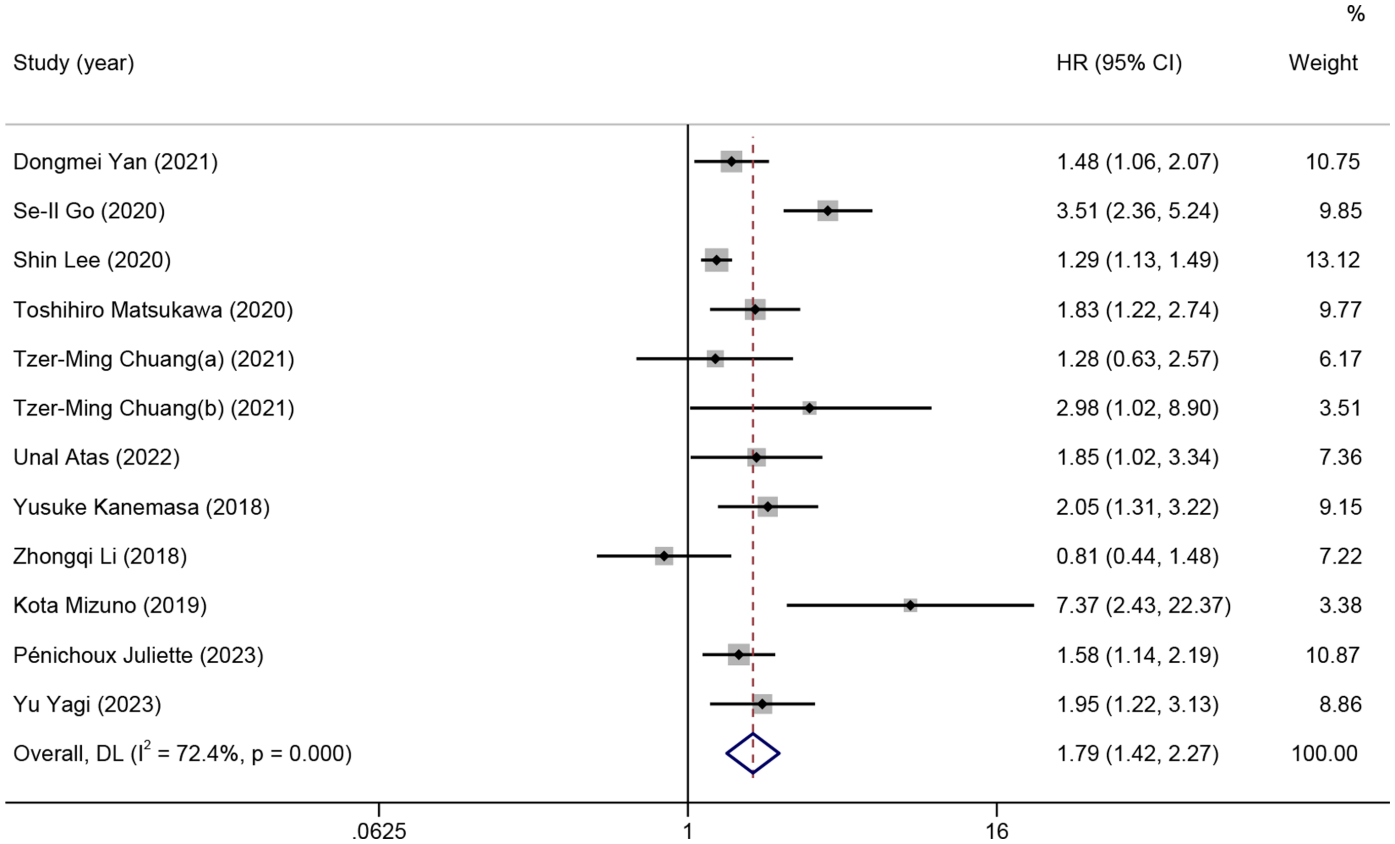
The forest plot for the association between GNRI and overall survival in patients with lymphoma. The boxes and the horizontal lines through the boxes illustrate the estimated hazard ratios and the length of the confidence interval. The diamond and horizontal line through the diamond show the pooled hazard ratio and confidence interval.

Eleven studies and 12 comparisons were included in the meta-analysis on the predictive value of GNRI on OS for lymphoma. Patients with low GNRI had shorter OS than those with high GNRI (HR = 1.79; 95% CI = 1.42–2.27, *p* < 0.01), with high heterogeneity (*I*^2^ = 72.4%; Figure 4). Meta-regression analyzes showed that no significant part of the variance could be explained by confounders in the heterogeneity including sex, age, stage, ECGO PS, B symptoms, LDH, country, cut-off value, the number of participants, and median OS.

### 3.6. Publication bias

**Figure 5:**
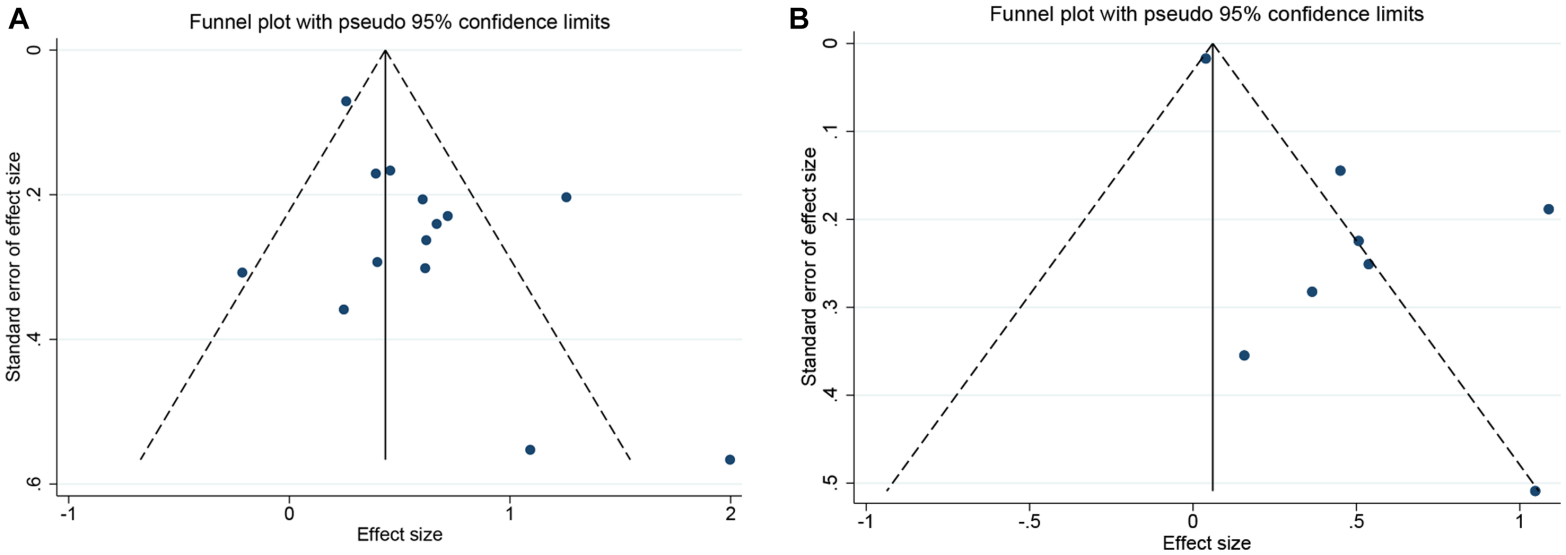
The funnel plot of meta-analysis on survival. **(A)** OS **(B)** PFS.

**Figure 6:**
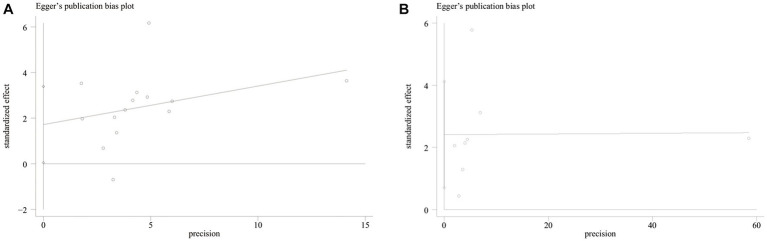
The Egger’s publication bias plot of meta-analysis on survival. **(A)** OS **(B)** PFS.

Both funnel plot (Figure 5) and Egger’s publication bias plot (Figure 6) were used to evaluate the potential publication bias for the association between GNRI and OS/PFS. Significant publication bias was detected by the Egger’s test (Egger’s test for OS: *p* = 0.045; Egger’s test for PFS: *p* = 0.014).

### 3.7. Sensitivity analysis

**Figure 7:**
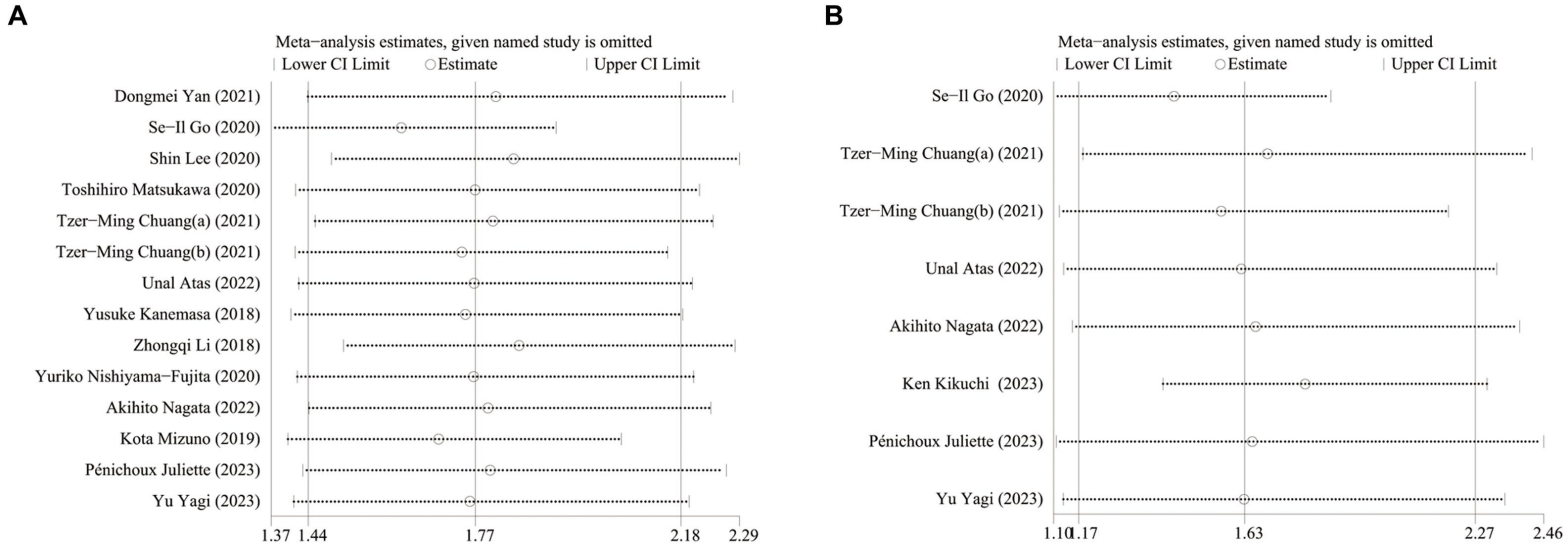
Sensitivity analysis of pooled HR for survival time and GNRI. **(A)** OS **(B)** PFS.

To verify the robustness of the results, a sensitivity analysis was performed. The sensitivity analysis results showed that the overall pooled data of OS and PFS did not change significantly when either included study was omitted (Figure 7).

## 4. Discussion

Since GNRI’s prognostic role has been demonstrated in HMs research, we conducted the meta-analysis to systematically evaluate the relationship between the GNRI and the clinical outcome in patients with HMs. The finding of our meta-analysis showed that low GNRI was associated with shorter OS and PFS. However, the negative effect of low GNRI on the survival was not significant in the subgroup analysis stratified by country. No significant benefit on PFS was not observed in the subgroup analysis based on cut-off value less than 98 and patients with non-DLBCL.

GNRI is an easily obtained, objective simple, efficient, and applicable tool to evaluate nutrition statues in the clinical practice. Currently, nutritional status in hospitalized patients is generally evaluated by systematic nutritional assessment methods, such as subjective global assessment (SGA), malnutrition universal screening tool (MUST), mini nutritional assessment (MNA) and Global Leadership Initiative on Malnutrition (GLIM). These measures include several components, such as weight loss history, anthropometry, and dietary intake change (42–45). In some cases, these approaches are difficult to implement completely. Previous studies have attempted to compare systematic nutritional assessment methods with GNRI. In a study conducted in Egypt, GNRI demonstrated a higher prognostic value for malnutrition-related clinical outcomes in hospitalized patients than MNA (46). The relationship between GNRI and SGA was also well-defined in elderly patients on hemodialysis (47). For GNRI, the serum albumin level can be easily obtained by routine examination of peripheral blood. As problems occurred in measuring the usual bodyweight of the elderly, the GNRI index replaces usual body weight with the ideal body weight (48). In addition, low GNRI predicted poor outcome in many cancers. In Wang’s study, GNRI was identified as a significant prognostic factor for OS and PFS in cervical cancer (49). A meta-analysis with 11 studies also showed that GNRI has good prognostic ability on OS (HR 1.96) in patients with non-small cell lung cancer (50). Our results demonstrated that pretreatment GNRI was an independent prognostic risk factor for patients with HMs.

To our knowledge, the mechanisms underlying linking between GNRI and cancer prognosis could be explained as follows. Firstly, GNRI index directly reflected nutritional status in hospitalized patients. Malnutrition is common in patients with HMs (51, 52). Different from malnutrition caused by simply insufficient energy intake, one of the leading causes of cancer-associated malnutrition is metabolic derangements caused by tumors (53). In addition, chemotherapy-induced nausea, vomiting symptoms, and decreased appetite further lead to a decrease in food intake, exacerbating malnutrition severity (54). A prospective multicenter cohort study has found that the pretreatment malnutrition based on GLIM criteria led to an increase in mortality for patients with non-Hodgkin’s lymphoma (52) and even affected the efficacy of immunotherapy (14). Malnourished patients with HMs are also at high risk of sarcopenia. Sarcopenic patients faced higher chemotherapy toxicity and poorer tolerance to oncological treatments (55). Recent meta-analyzes have also suggested that sarcopenia predicts impaired OS in patients with DLBCL (56). Cancer cachexia refers to weight loss due to sarcopenia and cancer-related inflammation (57). Cancer cachexia has also been proven to affect the clinical outcomes in patients with HMs negatively (58). Secondly, GNRI is also considered an immune nutritional parameter because albumin is closely related to nutrition status and inflammation. Decreased serum albumin levels are the result of inflammation and malnutrition (59). As a nutritional parameter, serum albumin level is a potent predictor for clinical outcomes in patients with acute myeloid leukemia and lymphoma (60, 61).

Another critical feature of albumin is its capability to mediate inflammation. Albumin could bind lipopolysaccharide, lipoteichoic acid, and peptidoglycan to cause inflammation through Toll-like receptor 4 (62). The serum albumin level could be regulated by inflammatory cytokines such as interleukin-1, interleukin-6, and tumor necrosis factor-alpha (63–65). The role of inflammation in the prognosis of HMs should not be ignored. In the included studies, different inflammatory conditions in different patients also influenced the predictive effect of GNRI. Studies have proven that the tumor microenvironment is largely orchestrated by inflammatory cells (66). Local immune response and systemic inflammation both promote the development of tumors and contribute to cancer migration. Cancer-related inflammation also regulates the efficacy of anti-cancer therapies (67, 68), slows the clearance of anticancer drugs and increases the side effects of treatment (69). Although GNRI also reflects the level of inflammation in the body to a certain extent, it is not enough to reflect the level of systemic inflammation in the body. Unfortunately, extracting relevant information for further analysis in meta-analysis is impossible. Studies have proven that the tumor microenvironment is largely orchestrated by inflammatory cells (66). In addition, malnutrition was also associated with inflammation. Weight loss (the so-called B symptoms) was also recognized as a sustained and systemic reaction to cancer (70). Cancer cachexia syndrome was considered as the most extreme result of systemic inflammation (71, 72). The inflammation may be another explanation for predictive role of GNRI on prognosis in HMs.

Notably, we did not observe differences in OS/PFS between low and high GNRI in our meta-analysis results in Chinese patients with HMs. It may be explained by the different study population selected in the included studies. In Li’s study, patients with low GNRI had short survival time, but the significance did not remain after adjusting NCCN- international prognostic index in the multivariate analyzes (30). The situation is less straightforward for studies using a GNRI cutoff less than 98. There was no significant difference in PFS, which suggested that the results may be influenced by the different cutoff values. Different from the previous two published meta-analyzes (31, 32), our study provides more evidence and information on the role of GNRI in predicting PFS in HMs.

Several other articles that explored the predictive values of GNRI cannot be ignored. One study reported that GNRI was significantly associated with ICU mortality for HM patients with acute respiratory failure treated in ICU (73). In Takuji Matsuo’s study, GNRI score < 82 correlated with poor 5-year OS (74). Another study also demonstrated statistical differences in GNRI values between patients who completed complete six or eight cycles of the standard regimens and those who did not (75).

Our meta-analysis is not free of limitations. First, most of the included studies were retrospective. Second, the potential heterogeneity among articles would lead to bias. There was considerable heterogeneity in the meta-analysis as a result of using different GNRI cut-off values. The GNRI cut-off value varied between 92 to 106.3 for OS. While, no moderators reached statistical significance in our meta-regression for lymphoma. Unfortunately, other moderators such as BMI, hemoglobin, extranodal involvement and CNS/liver/bone marrow invasion cannot be obtained from all the included studies. These factors could be significant parts of the variance in the heterogeneity. Third, eleven of the fourteen included studies were conducted in East Asia countries, which may affect the accuracy of the results and cause bias. In addition, the distribution of pathological types, treatment and prognosis of HMs are also different in different regions. Therefore, we were unable to make a partial generalization with limited information. Further research needs to confirm the prognostic effect of GNRI in patients with HMs from other countries. Fourth, the outcome reported in one article was TTF, which we also incorporated in the meta-analysis of PFS. It would cause bias. Fifth, one study included patients with myelodysplastic syndromes in addition to leukemia, considering that myelodysplastic syndromes frequently progress to acute myeloid leukemia, and the American Cancer Society also considered myelodysplastic syndromes as a type of cancer, so we included this study in the meta-analysis. This may also contribute to heterogeneity among studies.

## 5. Conclusion

In summary, for patients with HMs, Low GNRI predicted shorter OS and PFS. Identifying the best cut-off value for GNRI applied to patients with different features of HMs and prospective studies to validate the prognostic significance of GNRI in patients with HMs are areas for further research.

## Data availability statement

The original contributions presented in the study are included in the article/Supplementary material, further inquiries can be directed to the corresponding authors.

## Author contributions

QY: Conceptualization, Formal analysis, Writing – original draft. MT: Formal analysis, Writing – review & editing. GP: Data curation, Formal analysis, Writing – review & editing. YJ: Writing – review & editing. XJ: Conceptualization, Formal analysis, Writing – original draft.
