# Supplementary material for: Geriatric nutritional risk index as a predictor of prognosis in hematologic malignancies: a systematic review and meta-analysis
Source: Front Nutr. 2023 Oct 24;10:1274592. doi: 10.3389/fnut.2023.1274592 (PMC10627839; doi:10.3389/fnut.2023.1274592)
Supplement: Supplementary file 1 [file Data_Sheet_1.docx]

Supplementary Material

**1 Supplementary Tables**

| author/year | Study Participation | Study Attrition | Prognostic Factor Measurement | Outcome Measurement | Study Confounding | Statistical Analysis and Reporting | Overall Risk of Bias |
| --- | --- | --- | --- | --- | --- | --- | --- |
| Dongmei Yan 2021 | M | M | L | M | M | L | M |
| Se-Il Go 2020 | M | L | L | L | L | L | L |
| Shin Lee 2021 | L | L | L | L | L | M | L |
| Toshihiro Matsukawa 2020 | L | L | L | L | M | L | L |
| Tzer-Ming Chuang 2021 | L | L | L | M | M | L | M |
| Unal Atas 2022 | M | M | L | M | H | L | M |
| Yusuke Kanemasa 2018 | M | M | L | M | M | L | M |
| Zhongqi Li 2018 | M | M | M | H | H | L | H |
| Yuriko Nishiyama-Fujita 2020 | M | M | L | M | H | M | M |
| Akihito Nagata 2022 | M | M | L | M | L | L | M |
| Kota Mizuno 2019 | M | L | L | L | M | L | M |
| Ken Kikuchi 2023 | M | M | L | L | M | M | M |
| Pénichoux Juliette 2023 | M | M | L | L | L | L | M |
| Yu Yagi 2023 | M | M | L | L | L | M | M |

Table S1. Quality assessment of individual studies using the QUIPS instrument.

Note: L: low risk; M: moderate risk; H: high risk

Table S2. Newcastle–Ottawa quality assessment Scale appraisal for case-control and cohort study

| Study/Year | Selection | Comparability | Outcome/Exposure | Score |
| --- | --- | --- | --- | --- |
| Dongmei Yan 2021 | ★☆☆★ | ★★ | ★★☆ | 5 |
| Se-Il Go 2020 | ★★☆★ | ★★ | ☆★★ | 7 |
| Shin Lee 2021 | ★☆★★ | ★★ | ★★★ | 8 |
| Toshihiro Matsukawa 2020 | ★☆★★ | ★★ | ★★☆ | 7 |
| Tzer-Ming Chuang 2021 | ★☆☆★ | ★☆ | ★★☆ | 5 |
| Unal Atas 2022 | ★★☆★ | ☆☆ | ★★☆ | 5 |
| Yusuke Kanemasa 2018 | ★★☆★ | ★☆ | ★★☆ | 6 |
| Zhongqi Li 2018 | ★☆☆★ | ★☆ | ★★☆ | 5 |
| Yuriko Nishiyama-Fujita 2020 | ★☆☆★ | ☆☆ | ★★☆ | 4 |
| Akihito Nagata 2022 | ★★☆★ | ☆★ | ★★☆ | 6 |
| Kota Mizuno 2019 | ★☆☆★ | ☆☆ | ☆★☆ | 3 |
| Ken Kikuchi 2023 | ★☆☆★ | ★☆ | ★★☆ | 5 |
| Pénichoux Juliette, 2023 | ★☆☆★ | ★☆ | ☆★☆ | 4 |
| Yu Yagi 2023 | ★☆☆★ | ★☆ | ★★☆ | 5 |
